# In Silico Screening of Inhibitors of the Venezuelan Equine Encephalitis Virus Nonstructural Protein 2 Cysteine Protease

**DOI:** 10.3390/v15071503

**Published:** 2023-07-04

**Authors:** Xin Hu, Elaine Morazzani, Jaimee R. Compton, Moeshia Harmon, Veronica Soloveva, Pamela J. Glass, Andres Dulcey Garcia, Juan J. Marugan, Patricia M. Legler

**Affiliations:** 1 National Center for Advancing Translational Sciences (NCATS), Rockville, MD 20850, USA; xin.hu@nih.gov (X.H.); andres.dulcey@nih.gov (A.D.G.); maruganj@mail.nih.gov (J.J.M.); 2 General Dynamics Information Technology, Falls Church, VA 22042, USA; emorazza@vt.edu; 3 Center for Bio/Molecular Science and Engineering (CBMSE), Naval Research Laboratory, Washington, DC 20375, USA; jaimee.compton@nrl.navy.mil; 4 Department of Chemistry and Biochemistry, Jackson State University, Jackson, MS 39217, USA; harmonmoeshia08@gmail.com; 5 United States Army Medical Research Institute of Infectious Diseases, Frederick, MD 21702, USA; veronica.soloveva.ctr@mail.mil (V.S.); pamela.j.glass.civ@health.mil (P.J.G.)

**Keywords:** alphavirus, nsP2 cysteine protease, irreversible, reversible, inhibitor, oxindole, epoxide, antiviral drug target

## Abstract

The Venezuelan equine encephalitis virus (VEEV) nonstructural protein 2 (nsP2) cysteine protease (EC 3.4.22.B79) is essential for viral replication. High throughput in silico/in vitro screening using a focused set of known cysteine protease inhibitors identified two epoxysuccinyl prodrugs, E64d and CA074 methyl ester (CA074me) and a reversible oxindole inhibitor. Here, we determined the X-ray crystal structure of the CA074-inhibited nsP2 protease and compared it with our E64d-inhibited structure. We found that the two inhibitors occupy different locations in the protease. We designed hybrid inhibitors with improved potency. Virus yield reduction assays confirmed that the viral titer was reduced by >5 logs with CA074me. Cell-based assays showed reductions in viral replication for CHIKV, VEEV, and WEEV, and weaker inhibition of EEEV by the hybrid inhibitors. The most potent was NCGC00488909-01 which had an EC_50_ of 1.76 µM in VEEV-Trd-infected cells; the second most potent was NCGC00484087 with an EC_50_ = 7.90 µM. Other compounds from the NCATS libraries such as the H1 antihistamine oxatomide (>5-log reduction), emetine, amsacrine an intercalator (NCGC0015113), MLS003116111-01, NCGC00247785-13, and MLS00699295-01 were found to effectively reduce VEEV viral replication in plaque assays. Kinetic methods demonstrated time-dependent inhibition by the hybrid inhibitors of the protease with NCGC00488909-01 (K_i_ = 3 µM) and NCGC00484087 (K_i_ = 5 µM). Rates of inactivation by CA074 in the presence of 6 mM CaCl_2_, MnCl_2_, or MgCl_2_ were measured with varying concentrations of inhibitor, Mg^2+^ and Mn^2+^ slightly enhanced inhibitor binding (3 to 6-fold). CA074 inhibited not only the VEEV nsP2 protease but also that of CHIKV and WEEV.

## 1. Introduction

The Venezuelan equine encephalitis virus (VEEV) can be transmitted by mosquitos. VEEV contains a single-stranded positive-sense RNA ((+)ssRNA) genome and is a member of the alphavirus genus. The highly infectious new world alphavirus was once studied as a potential biological weapon prior to the signing of the Biological Weapons Convention in 1972 [1]. VEEV viral particles can replicate to high titers and can be stably dried and aerosolized [2]. New world alphaviruses cause flu-like symptoms within equines and humans and, in more severe cases, encephalitis and death. The more deadly eastern equine encephalitis virus (EEEV) is highly similar to VEEV and is classified as a Select agent. Western equine encephalitis virus, WEEV, has a 10% case fatality rate [3]. The nonstructural polyproteins of WEEV and EEEV share ~64% sequence identity with that of VEEV. In the United States, the number of mosquito-borne EEEV infections reported to the Centers for Disease Control (CDC) has varied between 4 and 15 per year over the past decade. In 2019, a total of 38 cases of EEEV were reported in the US, of which 19 resulted in death [4]. Only ~1% of human VEEV cases result in lethal encephalitis, while 20–80% of equine cases result in death. VEEV can infect dendritic cells and macrophages in lymphoid tissues, but also neurons and astrocytes in the brain [5]. In survivors of EEEV infections, sequelae such as mental retardation, epilepsy, paralysis, deafness, and blindness can occur [6].

Currently, there are no licensed vaccines for alphaviruses; however, two vaccines are being used under investigational new drug (IND) status to protect lab workers and those at risk of exposure: TC-83 and C-84 [7]. TC-83 is a live attenuated vaccine strain that elicits protection in animals from subcutaneous and aerosol exposure. C-84 is a formalin-inactivated vaccine-derived TC-83 that protects against subcutaneous exposure [8,9].

Alphaviruses are Group IV (+)ssRNA viruses; their genomes are essentially messenger RNAs. Translation occurs early after entry into the cell and precedes replication of the genome. The alphaviral genome encodes a structural and nonstructural polyprotein (nsP). The nsP contains nsP1 (RNA capping), nsP2 (helicase, cysteine protease, and a SAM methyltransferase domain), nsP3 (macrodomain), and nsP4 (RNA-dependent RNA polymerase). The nsP2 cysteine protease (nsP2pro, EC 3.4.22.B79) cleaves the polyprotein at three sites to produce four functional replication proteins. The cleavage is essential to replication and the alphaviral nsP2 cysteine protease has been validated as a drug target by deletion and mutation [10].

Other reasons for inhibiting the nsP2 protease include inhibiting the cleavage of the host proteins. We previously showed that human tripartite motif containing protein 14 (TRIM14) could be cut by the VEEV nsP2 protease [11]. TRIM14 was proposed to be a component of the mitochondrial antiviral signaling protein (MAVS) signalosome [12]. The MAVS signaling cascade leads to the generation of proinflammatory cytokines and interferon (IFN). Thus, cleavage of this protein may short-circuit this cascade, in order to antagonize the production of IFN.

Viral protease cleavage sites can be found in several host proteins [13,14,15,16]. Evidence of human protein cleavage by (+)ssRNA viral proteases dates back to the 1980s [14,15]. Protease cleavage site sequences span ~6–8 amino acids. Short stretches of homologous host-pathogen sequences (SSHHPS) in the junctions between the nsP may have been acquired from reservoir species during RNA recombination events as the RNA-dependent RNA polymerase is thought to pause at various secondary structures during genome replication. The vast majority of host proteins shown to contain viral protease cleavage have been related to innate immunity and IFN antagonism; only within the past 20 years has evidence begun to emerge showing that the cleavage of some of these host proteins is related to viral pathogenesis [13,16,17,18].

In Group IV (+)ssRNA viruses, mutations are relatively infrequent when compared with retroviruses (Group VI) [19]. Recognition of key host protein sequences by these viral proteases may act as a source of selective pressure as IFN antagonism and suppression of the innate immune responses are important to the establishment of infection. For drug discovery, the selective pressure from the host may be beneficial, as the proteases should retain their substrate specificities. We showed earlier that the membrane-permeable cysteine protease inhibitor, CA074 methyl ester (CA074me), halted the cleavage of TRIM14, indicating that it could effectively inhibit the protease in cells [11]. Viral protease involvement in pathogenesis also suggests that protease inhibitors may be able to halt various aspects of the virus-induced phenotype (e.g., cardiomyopathy, respiratory effects, encephalitis, etc.).

One complication with cysteine proteases is their redox sensitivity; small molecule inhibitors with redox activity can be spuriously picked up during screening. Thiols are known to react with pan-assay interference compounds (PAINS) [20] and “hits” from traditional high throughput screening (HTS) of compound libraries are often enriched with these compounds. For viral encephalitis, blood–brain barrier (BBB) permeability is also important. Molecular properties can be constrained in silico to enrich compounds with a higher likelihood of BBB entry. In our previous work [3], we determined the structure of the VEEV nsP2 cysteine protease inhibited with E64d (PDB 5EZS). Herein, we report the structure of the CA074me-inhibited VEEV nsP2 and its effects on viral replication in cells. Notably, the two inhibitors occupy different locations in the protease. To exploit this, we designed hybrid inhibitors with improved potency. We also report our findings on drug repurposing candidates and show our cell-based assay data.

## 2. Materials and Methods

### 2.1. Materials

All general chemicals were purchased from Fisher Scientific and Sigma. Plasmid constructs were synthesized by Genscript USA, Inc. (Piscataway, NJ, USA). BugBuster was purchased from EMD Millipore Corp. (Billerica, MA, USA). Column resins and PD-10 gel filtration columns were purchased from Cytiva Inc. (Marlborough, MA, USA). EDTA-free protease inhibitor tablets were from Roche, Inc. (Mannheim, Germany). Black half-area Corning #3993 nonbinding surface 96-well plates were from Corning Inc. (Corning, NY, USA). Tris-HEPES acrylamide gels (8–16% gradient) and SDS-PAGE running buffer were from Thermo Scientific (Rockford, IL, USA). The JCSG + screen was from Qiagen (Valencia, CA, USA). The gel imager is from BioRad Inc. (Hercules, CA, USA).

### 2.2. Plasmid Constructs of FRET Substrates

A pet15b plasmid (Ampicillin^R^)-encoding cyan fluorescent protein (CFP), an nsP1/2 protease cleavage site VEEPTLEADVDLMLQEAGA↓GSVETP, and yellow fluorescent protein (YFP) in between the NdeI and XhoI cut sites was synthesized (Genscript Inc., Piscataway, NJ, USA). An N-terminal hexa-histidine tag preceded a thrombin cleavage site. The cleavage site is denoted by an arrow to demarcate the residues that are N-terminal of the scissile bond and residues that are C-terminal of the scissile bond. The CFP–YFP construct containing the VEEV nsP1/2 junction contained was used for testing inhibitors.

### 2.3. Plasmid Constructs of VEEV nsP2 Protease

To ensure purification of the reduced state of the VEEV nsP2 cysteine protease, the protease was fused to thioredoxin (Trx) and a hexa-histidine tag with a thrombin cleavage site in a pet32a vector. The His-tag was associated with thioredoxin to facilitate the removal of cut and uncut protein by a second nickel column purification step. The Trx-VEEV-nsP2 construct contains the protease and *S*-adenosyl-L-methionine-dependent RNA methyltransferase (SAM MTase) domains (residues 457–792).

### 2.4. Expression and Purification of the VEEV nsP2 Cysteine Protease

BL-21(DE3) pLysS *E.coli* were transformed with the Trx-VEEV-nsP2 plasmid. Luria Bertani (LB) broth (3–6 L) containing 50 µg/mL ampicillin and 25 µg/mL chloramphenicol was inoculated and grown to an OD_600_ of approximately 1.0 and induced with 0.3 mM isopropyl β-D-1-thiogalactopyranoside (IPTG) overnight at 17 °C. Cells were pelleted and lysed with lysis buffer (50 mM Tris pH 7.6, 500 mM NaCl, 35% BugBuster, 5% glycerol, 2 mM β-mercaptoethanol (BME), ~30 mg DNase, and lysozyme) and sonicated ten times for 15 s intervals in an ice bath. Lysates were clarified by centrifugation at 20,000× *g* for 30 min and loaded onto a nickel column equilibrated with 50 mM Tris pH 7.6, 500 mM NaCl, 2 mM BME, and 5% glycerol. The column was washed with a buffer containing 60 mM imidazole. Protein was eluted using the same buffer containing 300 mM imidazole. Protein was dialyzed with thrombin against 50 mM Tris pH 7.6, 250 mM NaCl, 5 mM DTT, 1 mM EDTA, and 5% glycerol, and then loaded onto an SP-Sepharose column equilibrated with 50 mM Tris pH 7.6, 0–1.25 M NaCl, 5% glycerol, and 5 mM DTT. The nsP2 protease elutes in the upper end of the gradient (750 mM NaCl), whereas bovine thrombin has a near-neutral pI. Removal of thrombin was confirmed by testing a C477A variant. To remove any contaminating metal ions for metal-binding experiments, the protein was treated with 50 mM EDTA and then loaded onto a 23 cm G-25 Sephadex column equilibrated with 50 mM HEPES pH 7.0 buffer prior to kinetic assays. Protein was concentrated, flash-frozen in liquid nitrogen, and stored at −80 °C. The buffer was exchanged with the corresponding assay buffer prior to all kinetic experiments using PD-10 columns. The Chikungunya virus (CHIKV) nsP2 protease was expressed and purified by the same method and the His-tag and maltose-binding protein (MBP) were not removed.

### 2.5. Expression and Purification of His-Tag Free FRET Protein Substrates

BL-21(DE3) *E.coli* were transformed with the plasmids encoding the substrates. LB/Amp (1.5 to 3.0 L) was inoculated and grown to an OD_600_ of approximately 1.0 and induced with 0.3 mM IPTG overnight with shaking at 17 °C. Cells were pelleted by centrifugation, lysed with lysis buffer (50 mM Tris pH 7.6, 500 mM NaCl, 35% BugBuster, 2 mM BME, 0.3 mg/mL lysozyme, and 1 EDTA-free protease inhibitor tablet), and briefly sonicated for 1 min in an ice bath. Lysates were clarified by centrifugation (20,500× *g* for 30 min at 4 °C) and loaded onto a nickel column equilibrated with 50 mM Tris pH 7.6, 500 mM NaCl, and 2 mM BME. The column was washed with the same buffer after loading, and with buffer containing 60 mM imidazole until the A280 returned to baseline. Protein was eluted with the same buffer containing 300 mM imidazole. The protein was dialyzed against 50 mM Tris pH 7.6 and 150 mM NaCl overnight at 4 °C. All substrates were produced in high yield (typical yields were 60–80 mg per liter of media) and could be readily concentrated to 9.0–10.5 mg/mL. The substrates were used for continuous and discontinuous assays. For metal-binding studies, the His-tag was removed by adding thrombin. After cleavage, 0.1 mg/mL 4-(2-aminoethyl)benzenesulfonyl fluoride (AEBSF) was added and the protein was reloaded onto a nickel-charged chelating Sepharose column. The cut protein was collected in the flow through. The protein was dialyzed against 50 mM Tris pH 7.6 and loaded onto a Q-Sepharose column. The protein was then eluted with a salt gradient and concentrated. The concentrated protein was treated with 50 mM EDTA and desalted on a 23 cm G-25 Sephadex column equilibrated with 50 mM HEPES pH 7.0.

### 2.6. Continuous FRET Assay

For measurement of steady-state kinetic parameters, the method described by Ruge, et al. was followed [21]. Cleavage of the CFP/YFP (Forster resonance energy transfer (FRET) substrates was monitored continuously at room temperature (23 ± 3 °C) using excitation/emission wavelengths of 434/470 nm and 434/527 nm and calculated emission ratios. The CFP/YFP V12 substrate contained the VEEV nsP2 cleavage site sequence: VEEPTLEADVDLMLQEAGA↓GSVETP. Data were collected with a SpectraMax M5 plate reader from Molecular Devices Inc. (San Jose, CA, USA). Enzyme concentrations of ≤1 µM and a substrate concentration range of 10–140 µM (8 different concentrations) were used. Data were collected in triplicate (50 µL reaction volumes) in half-area black low binding surface 96-well plates. Plates were read for 5–120 min at 0.5 to 2 min intervals. After the reads were completed, the plates were sealed with film and allowed to digest overnight at room temperature, 23 ± 3 °C, to obtain the emission ratio after complete cleavage of the substrate. The fraction of substrate cleaved, *f,* at each time point was then calculated from the emission ratios using the following equation:

$$f=\frac{\left[\frac{\left(\frac{ex434}{em527}\right)}{\left(\frac{ex434}{em470}\right)}-r_{uncut}\right]}{\left(r_{cut}-r_{uncut}\right)}$$

The nmols of substrate cleaved at each time point were calculated by multiplying *f* by the nmols of substrate at t = 0 (S_0_). The value of *r_uncut_* corresponds to the emission ratio measured in the absence of enzyme, and the value of *r_cut_* is the emission ratio measured when the substrate is fully cleaved. Initial velocities were calculated at each [S] concentration from the linear range (*f ≤* 20%). Plots of time vs. nmols were linearly fit for each [S] concentration, and initial velocities (v_o_) were obtained from the slopes of the lines. Rates of spontaneous hydrolysis were measured in the absence of an enzyme and were subtracted from the enzyme-catalyzed rates. Data were fit to the Michaelis–Menten equation, v_o_ = (V_max_ × [S])/(K_m_ + [S]) using Grafit (Erithricus Software Ltd., West Sussex, UK).

### 2.7. Discontinuous Gel-Based Assay

Reaction mixtures (2.6 µM nsP2-Trx, 30 µM CFP/YFP substrate, 1× PBS pH 7.4, and 5 mM DTT) were incubated overnight (~18 h) at room temperature (R.T. = 23 ± 3 °C). The reactions were run until >90% of the substrate was cleaved by the WT enzyme. Reactions were stopped by mixing with Laemelli buffer (1:1) and heating the samples for at least 3 min at ≥70 °C. Cleavage products (10 µL) were separated by SDS-PAGE in 12-well 8–16% gradient gels in running buffer (100 mM Tris, 100 mM HEPES, 3 mM SDS, and pH 8 ± 0.5) at 110 V for 50 min. The calculated molecular weight of the uncut FRET substrate containing a 25 amino acid cleavage sequence was 58 kDa, and 31 kDa, and 27 kDa for the cut CFP and YFP products. The molecular weight of the enzyme for the Trx-His-tagged enzyme was 52.208 kDa, and 38.29 kDa for the Tag-free enzyme. The bands were well separated in 8–16% gradient gels. Boiling of the samples was required to achieve the sharp banding pattern. Densitometry was done using the BioRad Gel Dock Imager software (BioRad Inc., Hercules, CA, USA).

The discontinuous gel assay using a final inhibitor concentration of 200 µM and 1× phosphate buffered saline pH 7.4 was run at R.T. overnight and used as the primary screen.

### 2.8. Time-Dependent Inhibition of VEEV nsP2 by CA074 and Its Derivatives

Progressive inhibition by CA074 was measured in the absence of substrate with at least 5 different concentrations of inhibitor dissolved in DMSO, essentially as described previously [22]. The K_i_ is the dissociation constant prior to the chemical step; k_2_ is the maximum rate constant for inhibition at saturating inhibitor concentration.

k_obs_ = k_2_/(1 + (K_i_/[I])) (1)

The apparent bimolecular rate constant for inhibition, k_i_, describes the rate of formation of the covalent E-I complex from free enzyme and inhibitor in the absence of substrate, and was calculated according to Equation (2).

k_i_ = k_2_/K_i_ (2)

Inhibition was carried out in 50 mM HEPES pH 7.0 at room temperature (22 ± 3 °C) in the presence or absence of metal ions.

### 2.9. Crystallization

VEEV nsP2 protease was inhibited with 0.5 mM CA074 in 50 mM HEPES pH 7.0, 30 mM CaCl_2_, and concentrated to 15.3 mg/mL. Hanging drops (1:1) containing the protein and precipitant (0.1 M Bicine pH 8.5, 20% PEG 6000, and 25% glycerol) were incubated at 17 °C. Diffraction data were collected on a Bruker Micro-STAR rotating anode equipped with Helios optics and a Bruker Platinum 135 CCD area detector. The structure was determined by molecular replacement and refined with Refmac [23], CNS [24], and Coot [25].

### 2.10. Cell-Based ELISA Assays

ATCC-Vero 76 cells were seeded at 40,000 cells/well in 96-black well plates and incubated at 37 °C with 5% CO_2_ with EMEM (Invitrogen, Waltham, MA, USA) complete with 10% FBS + 1% L-Glutamine and Pen/Strep (EMEM-C) for 24 h. The compound was plated at varying dilutions (2×), then 50 µL the of compound was transferred to cell plates 24 h after seeding. The compound was added to three (3) cell plates, two (2) for virus infection evaluation, and one (1) for evaluation of cell viability via the Promega Cell Titer-Glo assay (Promega, Madison, WI, USA). At BSL-3, 50 μL of virus (VEEV Trinidad) diluted in MEM was added to corresponding infection plates. After adding the virus, the plates were incubated at 37 °C with 5% CO_2_ for 18–24 h. After incubation, plates were fixed in 10% buffered formalin and incubated at 4 °C for a minimum of 24 h before being removed from BSL-3 containment and transferred to BSL-2 for staining using an antibody cell-based ELISA detection assay [26].

### 2.11. Virus Yield Reduction Assay

Assays were performed essentially as described in our earlier work using A549 cells [27].

To evaluate VEEV inhibition, plates were blocked for 1 h at room temperature using a blocking buffer (1× PBS containing 3% BSA). After 1 h, plates were washed 3 times with PBS. Virus infection was detected in cells using the 1A4A monoclonal antibody (50 µL of 1:4000 dilution of 1 mg/mL) added to each well and incubated at room temperature for 2 h. Plates were washed 3 times with PBS; after washing, 65 µL of a 1:10,000 dilution of HRP goat antimouse secondary was added per well and incubated for 1 h at room temperature. After 1 h, plates were washed 3 times with PBS and 100 µL of Pierce HRP Pico Luminescent substrate (component A and B mixed) was added per well (Thermo Scientific, Waltham, MA, USA). Plates were examined for luminescence utilizing a Gemini EM provided by Molecular Devices Inc. The VEEV assay described evaluates the amount of virus spread in the cell layer per well for which a percent inhibition can be calculated.

Compound efficacy was evaluated in primary neuronal cells in 24-well plates infected with the VEEV Trinidad strain at an MOI = 1. After a 1 h incubation, cells were washed 2× with PBS to remove residual virus. Following the wash, fresh media containing 25–200 µM compound was added and cells were incubated at 37 °C, 5% CO_2_. At 24 hpi, supernatants were harvested and frozen at –70 °C for future analysis. Virus yield was quantitated by plaque assay on Vero 76 cells. Bafilomycin was included as a positive control and DMSO (same diluant as compounds) as a negative control. All samples were run in duplicate.

### 2.12. Virtual Screening and Focused Set

A focused library of cysteine protease inhibitors consisting of ~4000 small molecules was collected from a variety of resources including PubChem, Chemble, BindingDB, DrugBank, and Integrity. Virtual screening was performed using the AutoDock-based virtual screen program DOVIS. All small molecules were docked to the structure target of VEEV nsP2 (PDB code 2HWK [27]) and the top-ranked 500 hits were analyzed in terms of predicted binding free energies, the overall fit in the binding site, and binding interactions with key residues identified from the substrate binding models.

## 3. Results

### 3.1. In Silico and In Vitro Screening

The NCATS compound library contains >350,000 compounds. Hits were identified from a focused set of known cysteine protease inhibitors and confirmed using a VEEV nsP2 protease assay. High-throughput library screening, while more extensive, often produces a large number of nonspecific hits for cysteine proteases. Cysteine proteases are sensitive to oxidation and reducing agents are typically included in storage and assay buffers to keep the enzymes active. Reducing agents, such as DTT, can nonspecifically react with some compounds, in particular with covalent inhibitors. Some compounds interfere with the fluorescence of the substrate; thus, the discontinuous SDS-PAGE assay in 1× PBS pH 7.4 without a reducing agent was used as the primary screening assay.

A variety of predictive methods to enhance the selection of BBB-permeable compounds in HTS have been reported and incorporated into the software. To minimize false positives and increase the likelihood of finding a BBB-permeable compound we limited the molecular weight (MW) (<500 g/mol), topological polar surface area (TPSA < 90 Å^2^), and number of H-bond donor/acceptors. This allowed us to identify low MW inhibitors that were still highly effective.

Using a focused in silico/in vitro screen to identify novel chemotypes, we tested 534 compounds in vitro at a single concentration (200 µM) in the SDS-PAGE gel-based assay. Enzyme, substrate, and inhibitor were coincubated at room temperature for 16 to 17 h in 1× PBS pH 7.4 without DTT. The reactions were boiled for 3 min and then separated by SDS-PAGE. Only 63 of the 534 compounds (11.8% hit rate) tested were able to inhibit the enzyme for this period of time; the incubation time was selected to be on par with the cell-based assays using live virus. The 11.8% hit rate of the combined in silico/in vitro screening is higher than what would be expected from traditional HTS. Traditional HTS hit rates as low as 0.01% have been reported [28]. Compounds that appeared to denature or aggregate the protein substrate were also identifiable in the gel assay; these compounds typically reduced the amount of protein that entered into the gel and gave less intense bands or diffuse bands in the gel.

Three chemotypes were identified from the gel-based assay: chloromethylketones, oxindoles, and epoxides. Selected hits, derivatives, and controls (73 compounds) were tested in a live virus cell-based assay with VEEV. The two chloromethyl ketones, MLS001172952 and MLS001178292, did not demonstrate significant antiviral activity against VEEV-Trd in cell-based assays at 50 µM and MLS001178292 was cytotoxic. The oxindoles were effective inhibitors in the in vitro protease assays (Figure A2), but failed to display significant antiviral activity against VEEV-Trd. Two compounds showed inhibition at <15 µM in cell-based assays NCGC00163431-03 and NCGC00018306-02. NCGC00163431-03 is a stereoisomer of CA074me and was a potent inhibitor (82.9% inhibition at 1.562 µM) in VEEV-infected HeLa cells. It only demonstrated toxicity at 100 µM. Thus, derivatives of CA074me and E64d were synthesized and analyzed.

### 3.2. CA074me, but Not E64d, Effectively Reduces Virus Yield in A549 Cells

**Figure 1:**
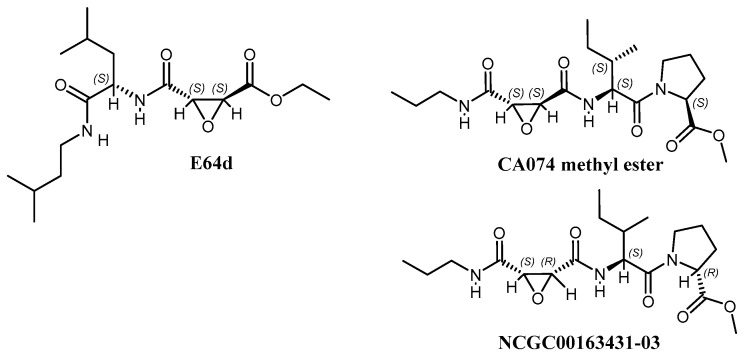
Structures of E64d, CA074 methyl ester, and NCGC00163431-03 inhibitors.

CA074 and E64 are covalent inhibitors of cysteine proteases in vitro (Figure 1) such as cathepsin B and L [29]. Cathepsin B is thought to be required for NLRP3 inflammasome activation [30]; thus, the overlap in inhibitor specificities is not necessarily adverse. The methyl esters have enhanced membrane permeability and can inhibit these proteases in cells [29]. Once inside the cell, these esters are hydrolyzed; thus, they are considered prodrugs. CA074 is a derivative of E64. Weak binding covalent inhibitors can still effectively inhibit an enzyme over time. We found that A549 cells infected with VEEV, EEEV, WEEV, or CHIKV that CA074me showed clear reductions in plaque assays relative to the DMSO control (Table 1). For VEEV, a nearly 6-log reduction in plaque-forming units (PFU) was observed with 100 µM CA074me. For EEEV, WEEV, and CHIKV, only a 2-log reduction in PFU was observed with 100 µM CA074me.

In our previous structure of VEEV, nsP2 inhibited with E64d (PDB 5EZS) [3]. We found that E64d, but not E64c was able to inhibit the nsP2 protease in vitro. This result is also consistent with the lack of inhibition by E64d in virus-infected cells (Table 1). Only minor reductions in titers were observed in VEEV- and CHIKV-infected A549 cells and no effect was observed in EEEV- and WEEV-infected cells (Table 1).

### 3.3. CA074me Effectively Reduces Virus Yield in Primary Neuronal Cells

**Figure 2:**
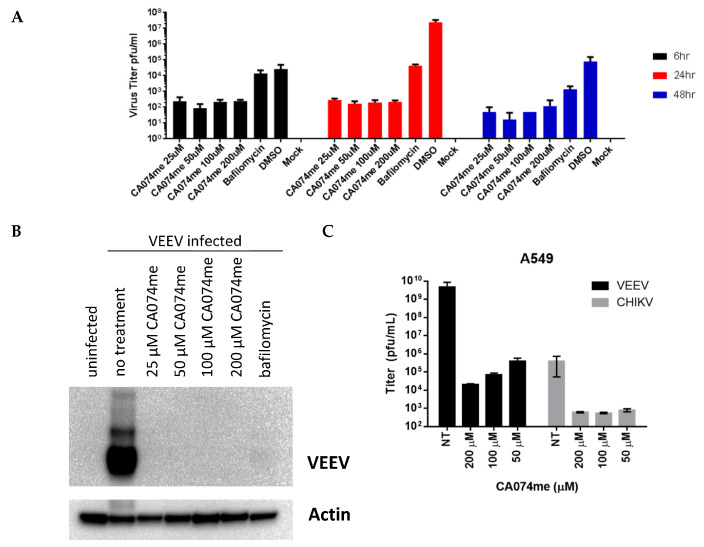
(**A**) Efficacy of CA074me in primary neuronal cells infected with VEEV-Trd at an MOI = 1. (**B**) Efficacy of CA074me in A549 cells. (**C**) Plaque assays show significant reductions in PFU.

Primary neuronal cells were also infected with VEEV Trinidad at an MOI = 1 for 1 h and then washed twice to remove residual virus (Figure 2). Following the wash, the compound was added and, after 24 hpi (hours post infection), the supernatants were harvested at different time points. Significant reductions in plaque forming units (PFU) (~5-log reduction at 24 h) were apparent as early as 6 h and still at 48 h.

### 3.4. X-ray Crystal Structure of CA074 Bound to the VEEV nsP2 Cysteine Protease

We previously examined a well-known mechanism-based irreversible epoxide inhibitor of cysteine proteases E64d. E64d is a membrane permeable prodrug. In the cell, it is hydrolyzed by esterases to produce the negatively charged E64c acid. Many cysteine proteases are readily inhibited by E64d and E64c; papain is inhibited by nanomolar concentrations of E64c [3]. E64c also inhibits cathepsin B, H, and L. Using the method of Kitz and Wilson [22], the maximal rate constant for inactivation k_2_ = 0.10 ± 0.06 1/min, and the dissociation constant, K_i_ = 40 ± 30 µM, were measured for the time-dependent inhibition of VEEV nsP2 protease. In discontinuous assays (SDS-PAGE gels) 17 h incubation of E + I + S at room temperature (RT, 23 ± 3 °C) without DTT led to complete inhibition using 200 µM E64d; in contrast, only partial inhibition was observed using 200 µM E64c. In cell-based assays, both E64d and E64c were ineffective; however, CA074me was effective in plaque assays (Table 1) but had a high K_i_ = 1200 ± 500 µM in vitro. E64c contains a carboxylic acid while E64d contains an ester. The negatively charged oxygen of the acid is near His-276-Nδ1 (2.5 Å) and Cys-SH (3.1 Å); these interactions may reduce the nucleophilicity of the Cys and disfavor the formation of the thiolate or lock the His-546 in a catalytically unproductive conformation. The mobility of the His in papain has been thought to play an important role in catalysis and may also be in the VEEV nsP2 protease.

**Figure 3:**
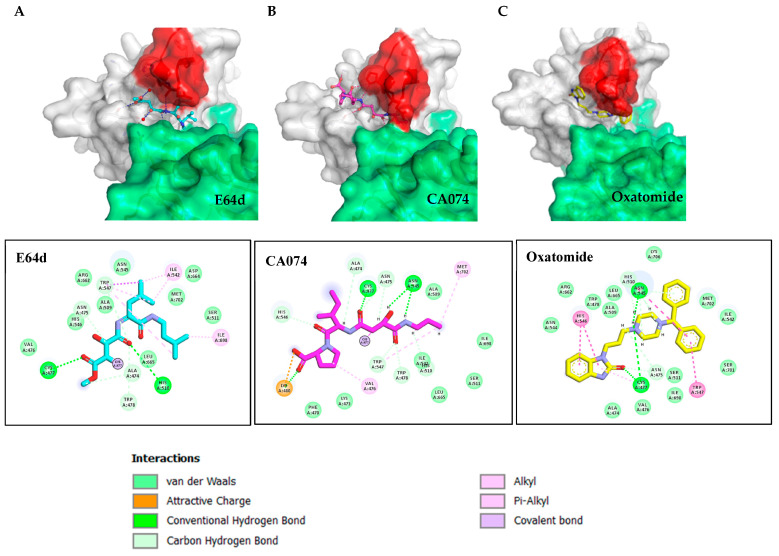
Binding sites of E64d versus CA074 (**A**) Structure of E64d (PDB 5EZS). In green is the SAM MTase domain (606–787); in white is the cysteine protease domain. In cyan is the E64d inhibitor covalently attached to the nucleophilic Cys-477. (**B**) Structure of the CA074-inhibited enzyme (PDB 8T8N). In magenta is the CA074 inhibitor covalently attached to the Cys-477. In the loop, the side chain of N545 changes and moves closer to the SAM MTase domain. (**C**) Docked model of oxatomide in the VEEV nsP2 cysteine protease. Below each structure is the contact residue map. The interactions are color coded as shown.

To examine the differences in inhibitor specificity, the CA074-inhibited VEEV nsP2 protease was crystallized (Figure 3). Data statistics for the structure are shown in Table 2. While the compounds appear similar, the regions occupied by the inhibitors differ (Figure 3B). In PDB 8T8N, the carboxylic acid moiety of CA074 is approximately 3.5 Å from Lys-480. The histidine of the catalytic dyad rotates towards the inhibitor when the active site becomes occupied. CA074 makes 8 h bonds to the enzyme. Interestingly, we tested a stereoisomer of CA074 (Figure 1), NCGC00163431-03 (CID 73265357). This compound showed inhibitory activity (83%) at 1.562 µM in VEEV-infected HeLa cells and cytotoxicity only at 100 µM, suggesting a better alignment between the nucleophilic cysteine of the viral protease and the C6 carbon of the inhibitor. E64d (PDB 5EZS) only makes 4 h bonds to the enzyme and two h bonds to water molecules.

### 3.5. Effects of Divalent Metal Ions on Covalent Inhibition

**Figure 4:**
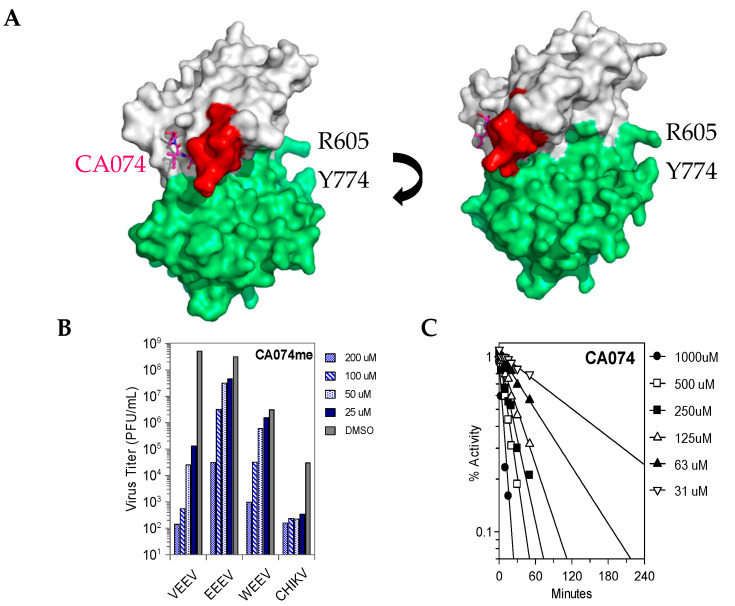
Structure of the CA074-inhibited VEEV nsP2 cysteine protease. (**A**) On the right is a side view of the inhibited enzyme. The surface shows a channel at the interface of the two domains. In white is the papain-like protease domain and in green the SAM MTase domain. In red is the β-hairpin which clamps down on the substrate. The peptide substrate may thread through the channel formed by the β-hairpin, R605, and Y774. In a structure of an N475A variant of the protease (PDB 6BCM), the N-terminus is found flipped into this channel. (**B**) Virus Yield Reduction (plaque) assays performed in vero cells showing that CA074me inhibits viral replication for all 4 viruses. The most significant reductions occurred in the VEEV- and CHIKV-infected cells. (**C**) Protease assay showing time-dependent inhibition of the VEEV nsP2 Cys-protease in the absence of metal ions. At 3 h, 50% inhibition is achieved with the lowest concentration; note that the cell-based assays are run for ~17 h and the replication time is ~3 h for VEEV.

CA074 is a time-dependent inhibitor (Figure 4). Divalent metal ions were tested for their effects on covalent inhibition (Table 3). Mn^2+^ and Mg^2+^ reduced the maximal rate constant of inactivation and reduced the K_i_ values three- to sixfold; however, no net effect on inhibitor specificity was observed (k_2_/K_i_). Mg^2+^ bound the V12 substrate weakly with a measured K_d_ = 2.10 ± 0.1 mM. Calcium was also bound to the V12 substrate with a K_d_ = 1.9 ± 0.1 mM.

### 3.6. Synthesized Derivatives of Hybrid CA074/E64d

**Figure 5:**
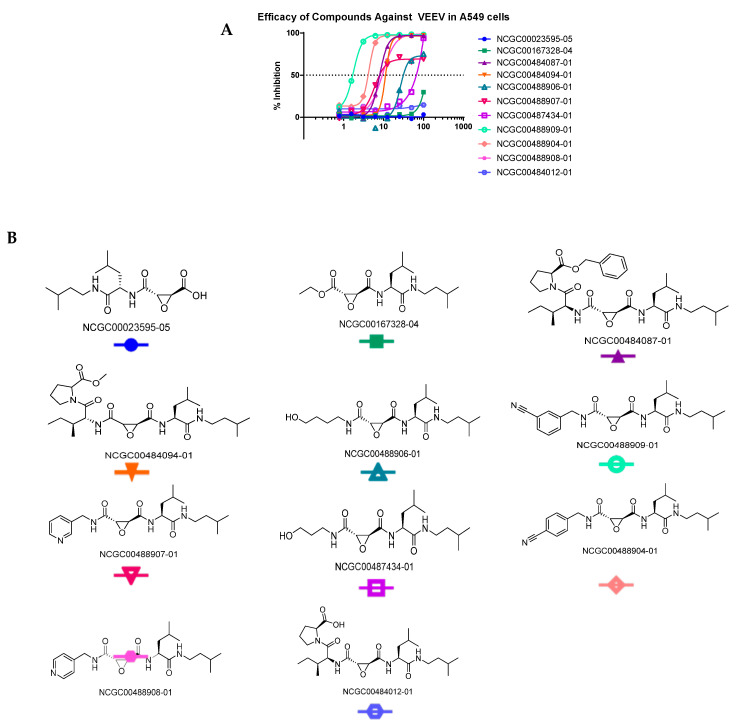
Hybrid inhibitors of the VEEV nsP2 cysteine protease. (**A**) The compounds were tested in A549 cells with VEEV. The most potent was NCGC00488909-01. (**B**) The hybrid inhibitors were synthesized and deposited into the NCGC compound libraries.

**Figure A1:**
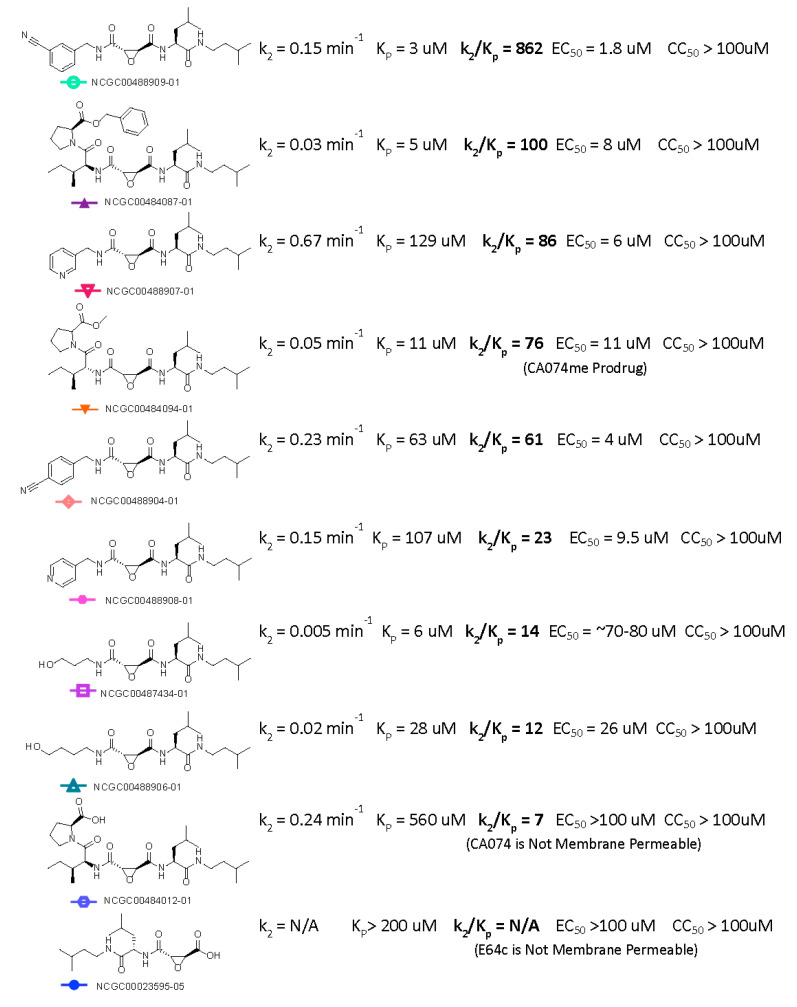
Kinetic parameters for inhibition for each synthesized compound.

**Figure A2:**
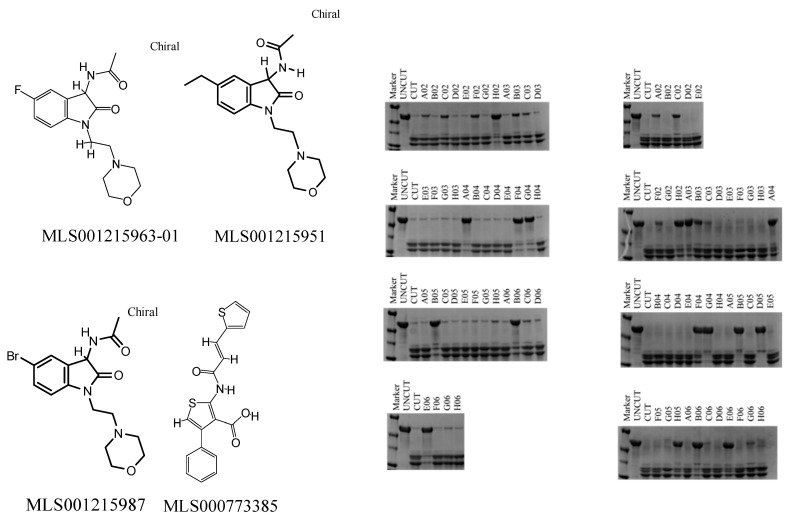
Oxindole compounds tested are shown in the table and the SDS-PAGE gel assays for the VEEV nsP2 protease are shown.

Since the two inhibitors occupied different regions of the substrate binding site (Figure 3) we synthesized hybrid inhibitors to exploit contacts in both regions (Figure 5 and Figure A1). These compounds potently inhibited the protease. NCGC00488909-01 was the most potent of the compounds tested. The in vitro kinetic data (k_2_/K_i_) correlated well with the efficacy of the compounds in cells suggesting minimal off-target inhibition (Table 4). NCGC00488909-01 had the highest k_2_/K_i_ value indicating the highest inhibitor specificity. Microsomal stability assays (Table 5 and Table A1) indicate that some of the epoxides are stable, whereas others are short lived.

### 3.7. Other Compounds Found in the NCATS Library with Anti-Alphaviral Activity in Primary Neurons

**Figure 6:**
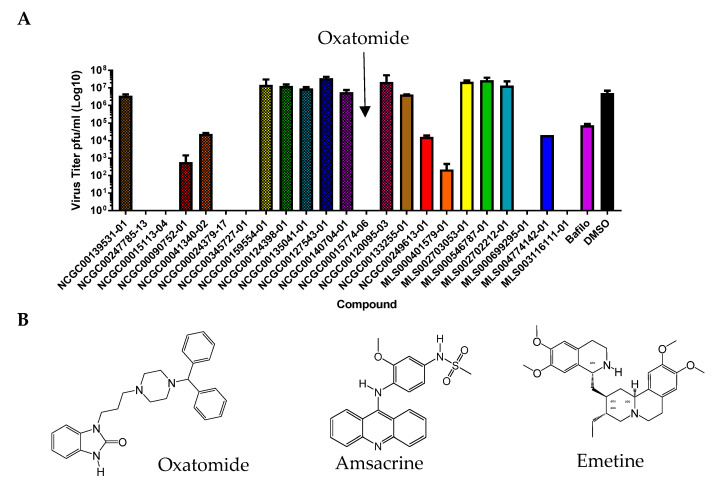
(**A**) Efficacy of other compounds such as oxatomide, emetine, and amsacrine in primary neuronal cells infected with VEEV Trinidad at an MOI = 1. Compounds were tested at 50 µM. (**B**) Compound structures.

We were able to identify other compounds with reported Group IV antiviral activity that were in the NCATS libraries. The mechanisms of action are unknown; thus, we selected and tested a small set in VEEV-Trd infected primary neurons (Figure 6). Among these, oxatomide an H1 antihistamine (NCGC00015774), emetine an inhibitor of protein synthesis (NCGC00024379), amsacrine an intercalator (NCGC0015113), MLS003116111-01, NCGC00247785-13, and MLS00699295-01 had notable antiviral activities in plaque assays. These compounds have been tested in humans. The H1 antihistamine mequitazine was given to 104 patients for the treatment of Chikungunya inflammation (arthralgia); using questionnaires, Deregnaucourt, et al. showed that joint pain was lessened [31]. Oxatomide was listed in their patent as a compound similar to mequitazine but the mechanism of action or antiviral effects (e.g., plaque assays) was not described. Here, we found antiviral activity associated with these three compounds in a plaque assay. We were also able to dock oxatomide into the VEEV nsP2 protease. Our data suggest that these three compounds may warrant further investigation for drug repurposing and mechanism of action studies in the future.

## 4. Discussion

Very few compounds have shown efficacy in cell-based assays against alphaviruses and only a few have been tested in vivo. Experimental compounds such as ML336 and BDGR-4 have shown efficacy in mice [32,33,34], but have not yet received FDA approval, and resistance to ML336 is obtainable in the lab.

Here, we examined covalent inhibitors of the VEEV nsP2 cysteine protease and showed that these compounds are potent inhibitors that have efficacy in vitro and in cells. Covalent inhibitors have long residency times in the active site and lower doses are possible. CA074 is a well-known inhibitor of cathepsin B; in our in vitro assays, we found that a stereoisomer of CA074 more potently inhibited the VEEV nsP2 viral protease, suggesting a strategy for specific inhibition. To date, only two structures of inhibitor-bound alphaviral enzymes exist in the PDB, that of the E64d-inhibited nsP2 protease (PDB 5EZS) and the CA074-inhibited protease (PDB 8T8N) described herein. Notably, these two inhibitors occupy the subsites which bind the most highly conserved residues of alphaviral protease substrates. Cathepsin B plays a role in inflammasome activation; thus, off-target effects may not be unfavorable in the case of viral encephalitis.

Cysteine proteases can be inhibited by reversible, irreversible, and reversible-covalent inhibitors (e.g., nitriles). Nitriles can reversibly react with the nucleophilic cysteine to mimic the oxyanion in the transition state. The H-bond acceptor and donor pattern of cysteine proteases are better matched with the nitrile intermediate than with a carbonyl or oxyanion. Interestingly, our most potent hybrid compound, NCGC00488909-01, contained both a nitrile and an epoxide.

In our prior work, we found that several host protein sequences could be cut by the VEEV nsP2 cysteine protease [11,13]. ADGRA2 (also known as TEM5 and GPR124) was predicted to be cut by proteases from eight neuroinvasive viruses by our sequence-to-symptom software for Group IV viruses. ADGRA2 is an orphan G-protein-coupled adhesion receptor that acts as a Wnt7 coactivator of β-catenin signaling [35]. The effect of the proteolytic cleavage of ADGRA2 is currently unknown, the predicted cleavage sites of the VEEV nsP2 cysteine protease are within regions that are predicted to be intracellular. ADGRA2 is also thought to contain a GPCR autoproteolysis-inducing (GAIN) domain in the extracellular portion of the protein. Conditional knockouts of ADGRA2 in mouse models of stroke and glioblastoma multiforme were shown to have compromised blood–brain barrier integrity and hemorrhages in the cerebrovasculature of the forebrain and ventral spinal cord [35,36]. Microhemorrhages in the brain have been observed in eastern equine encephalitis and may be related to the protease [37].

The inhibition of the VEEV nsP2 cysteine protease may result in two effects. First, it may inhibit polyprotein processing and viral replication. Second, it may inhibit the cleavage of host proteins. Many of the proteins targeted by viral proteases are involved in generating the innate immune responses; thus, protease inhibitors may alleviate the suppression of the innate immune responses by the virus. Cysteine protease inhibitors against Group IV viruses have proven to be effective; for example, PF-07321332 (Nirmatrelvir) from Pfizer, a component of Paxlovid™, is used to treat SARS-CoV-2.

We also identified approved and over-the-counter compounds, oxatomide, ementine, and amsacrine as antivirals against VEEV infection. Oxatomide is an interesting hit; this compound is an over-the-counter (oral drug) first-generation H1 antihistamine that causes drowsiness. Drowsiness is characteristic of first-generation H1 antihistamines (e.g., Benadryl). These compounds are known to cross the blood–brain barrier. We found that several oxidinoles had inhibitory effects on the VEEV nsP2 protease but that none were highly potent. Oxatomide is a benzimidazolinone and was a potent inhibitor in plaque assays. The H1 antihistamines have been recognized by others [31] for their apparent effects on Chikungunya infection in humans. However, antiviral effects were not previously demonstrated in the literature. Here, we show for the first time an effect on plaque-forming units using VEEV Trinidad. The nsP2 cysteine proteases of VEEV and CHIKV are very similar structurally. We were able to dock oxatomide into the substrate binding site of the VEEV nsP2 protease, suggesting that further investigation of this compound may be warranted, as very few options for the treatment of VEEV and EEEV infections are currently available.

## 5. Conclusions

Using a combined in silico/in vitro screening approach, we have identified compounds with antiviral activity against alphaviruses. Oxatomide, emetine, and amsacrine, among others, may be useful to further investigate for drug repurposing, as there are currently no approved drugs to treat alphaviral infections. We have also synthesized hybrid inhibitors based upon our crystal structures. These inhibitors take elements from both CA074 and E64d. The hybrid inhibitors displayed antiviral activity in cell-based assays against VEEV, CHIKV, WEEV, and EEEV.

## 6. Patents

A patent application has been submitted for the FRET substrates of the protease.

## Figures and Tables

**Table 1:** Efficacy of CA074me, but not E64d, in reducing Virus Yield in A549 cells. The DMSO control contains no inhibitor.

	AVERAGE TITER						
	CA074me				E64D		DMSO
[I]	200 µM	100 µM	50 µM	25µM	100 µM	50 µM	
VEEV	1.40 × 10^2^	5.49 × 10^2^	2.50 × 10^4^	1.33 × 10^5^	1.48 × 10^7^	4.17 × 10^7^	5.15 × 10^8^
EEEV	3.12 × 10^4^	3.20 × 10^6^	3.18 × 10^7^	4.57 × 10^7^	2.28 × 10^8^	2.27 × 10^8^	3.13 × 10^8^
WEEV	9.73 × 10^2^	3.17 × 10^4^	5.98 × 10^5^	1.54 × 10^6^	2.13 × 10^6^	2.16 × 10^6^	3.08 × 10^6^
CHIKV	1.58 × 10^2^	2.35 × 10^2^	2.23 × 10^2^	3.42 × 10^2^	2.70 × 10^3^	1.07 × 10^4^	2.99 × 10^4^
							
	STDEV						
	CA074me				E64D		DMSO
[I]	200 µM	100 µM	50 µM	25µM	100 µM	50 µM	
VEEV	7.5 × 10^1^	2.0 × 10^2^	5.3 × 10^3^	4.3 × 10^4^	5.4 × 10^6^	8.9 × 10^6^	9.5 × 10^7^
EEEV	7.3 × 10^3^	2.3 × 10^5^	1.5 × 10^6^	4.5 × 10^6^	9.6 × 10^7^	5.9 × 10^7^	4.3 × 10^7^
WEEV	1.8 × 10^2^	9.1 × 10^3^	2.2 × 10^5^	7.1 × 10^5^	9.9 × 10^5^	9.9 × 10^5^	1.1 × 10^6^
CHIKV	2.3 × 10^1^	1.3 × 10^1^	5.4 × 10^1^	6.4 × 10^1^	2.6 × 10^2^	9.2 × 10^2^	5.3 × 10^3^

**Table 2:** X-ray data statistics.

PDB ID	8T8N
Space group	P2(1)2(1)2(1)
Unit Cell Dimensions (Å)	60.76, 63.58, 86.28
Wavelength (Å)	1.54
Resolution Range (Å) ^a^	63.58–2.32 (2.42–2.32)
Unique Reflections	14,251 (1625)
R_sym_	0.051 (0.172)
I/σI	18.03 (6.08)
Completeness	94.6 (92.8)
Redundancy	13.35 (12.79)
**Refinement Statistics:**	
Resolution (Å)	51.19–2.32
No. of reflections	13,506
R_factor_	0.191
R_free_ ^b^	0.200 (5%)
Number of Atoms:	
Protein	2577
Solvent	136
Other	26
Average B-factors (Å^2^)	
Protein	19.9
Solvent	23.3
R.m.s.d. from ideal geometry:	
Bond lengths (Å)	0.006
Bond angles (degrees)	1.23
Ramachandran plot	
Most favored regions (%)	90.0%
Additional allowed regions (%)	10.0%
Generously allowed regions (%)	0.0%
Disallowed regions (%)	0.0%

^a^ Values in parentheses are for the outermost data shell. ^b^ R_free_ for test set and size of the test set as % total reflections in parentheses.

**Table 3:** Effects of metal ions on time-dependent inhibition of the VEEV nsP2 protease by CA074. Parameters were measured in 50 mM HEPES pH 7.0 at R.T. (23 ± 3 °C).

Metal	Inhibitor	K_i_ (µM)	k_2_ (min^−1^)	k_2_/K_i_ (µM^−1^min^−1^)
No Metal	CA074	1200 ± 500	0.23 ± 0.09	0.0002 ± 0.0001
MnCl_2_	CA074	410 ± 60	0.08 ± 0.03	0.0002 ± 0.0001
MgCl_2_	CA074	210 ± 90	0.04 ± 0.01	0.0002 ± 0.0001
CaCl_2_	CA074	2000 ± 1000	0.3 ± 0.2	0.0002 ± 0.0002
No Metal	E64d	40 ± 30	0.10 ± 0.06	0.002 ± 0.002

**Table 4:** Cell-based assay data on E64d and CA074me hybrid derivatives show that the hybrid derivatives are more effective than E64d or CA074me.

Compound ID	VEEV-Trinidad EC_50_ (µM)	VEEV-TC83 EC_50_ (µM)	VEEV nsP2 PROTEASE k_2_/K_i_ (M^−1^ s^−1^)	CHIKV EC_50_ (µM)	WEEV EC_50_ (µM)	EEEV EC_50_ (µM)	CC_50_ (µM)	CC_50_ (µM)
Cell Line	A549	HeLa	Protease	U2OS	HeLa	A549	HeLa	A549
NCGC00488909-01	1.76	1.48	862	9.85	5.02	43	>50	>50
NCGC00484087-01	7.90	2.17	100	(Not Tested)	5.74	13.23	>50	>50
NCGC00488907-01	5.97	5.22	86	25.92	8.76	<50	>50	>50
NCGC00488904-01	4.29	4.09	61	14.83	5.15	16.19	>50	>50
NCGC00488908-01	9.45	9.23	23	50.00	14.55	≥100	>50	>50
NCGC00488906-01	26.4	26.62	12	18.48	20.36	>100	>50	>50

**Table 5:** Pharmacological properties. Ideal compounds have a microsomal stability t_1/2_ > 30 min. For PAMPA (parallel artificial membrane permeability assays), compounds with P_app_ > 10 × 10^−6^ cm/s are considered to be highly permeable.

Compound ID	Microsomal Stability t_1/2_	PAMPA	Solubility
	Min	×10^−6^ cm/s	µg/mL
NCGC00484087-01	1.5	1089.1	4.1
NCGC00484012-01	>30	<1	>77
NCGC00484094-01	11.1	33	22
NCGC00488906-01	>30	ND	15.9
NCGC00488907-01	6.4	<4.3	ND
NCGC00488908-01	>30	7.6	>60

## Data Availability

The structure of the CA074-inhibited VEEV nsP2 cysteine protease has been deposited in the Protein Database Bank as PDB 8T8N.

## Appendix A

Results on other tested compounds are included in Appendix A.

**Registration ID**	**Well**	**Parent MW (g/mol)**	**IC50 (µM)**	**EC50 (µM)**	**CC50 (µM)**
MLS001179882-01	A02	292.7	>200	>50	>50
MLS001197204-01	A03	349.3	30 ± 20	>50	>50
MLS001215963-01	A04	321.3	16 ± 3	NT	
MLS001215939-01	A05	339.3			
MLS001215927-01	A06	325.3			
MLS001220062-01	B02	556.6			
MLS001198393-01	B03	442.7	>200	>16.67	>50
MLS001222475-01	B04	431.5			
MLS001177992-01	B05	303.7	NT	>50	>50
MLS001178292-01	B06	364.8	>200	>50	>50
MLS001172952-01	C02	383.8	>200	>50	>50
MLS001178294-01	C03	400.8			
MLS001097193-01	C04	343.8			
MLS001222477-01	C05	445.6			
MLS001173297-01	C06	318.8			
MLS000577775-01	D02	369.4			
MLS000553324-01	D03	475.6			
MLS000760808-01	D04	401.4			
MLS001030320-01	D05	281.2	>100	>50	>50
MLS001002335-01	D06	361.4			
MLS001224091-01	E02	387.8			
MLS000712897-01	E03	316.2			
MLS001097173-01	E04	323.3			
MLS000760597-01	E05	338.4			
MLS001215951-01	E06	331.4	18 ± 7	>50	>50
MLS000061991-01	F02	254.3			
MLS000712758-01	F03	270.2			
MLS001215987-01	F04	382.3	14 ± 3	>50	>50
MLS000711802-01	F05	271.7			
MLS000772304-01	F06	263.3			
MLS001097176-01	G02	393.2			
MLS001083053-01	G03	284.3			
MLS000877947-01	G04	292.7	>200	>50	>50
MLS000544331-01	G05	356.4			
MLS000773385-01	H02	355.4	15 ± 2	>50	>50
MLS001033759-01	H03	333.4			
MLS000717223-01	H04	515.6			
MLS001212815-01	H05	392.9	>200	>50	>50
NCGC00182390-04	G06	404.5			
NCGC00247785-05	H06	474.6			

**Table A1:** Microsomal stability and PAMPA assay results for selected compounds.

NCGC ID		Rat_SD/Microsomes t1/2 (m)	Permeability (PAMPA_Pion_Lipid × 10^−6^ cm/s)	Solubility (μg/mL)
NCGC00389066-01		ND	>2165	10.4
NCGC00484087-01		1.5	1089.1	4.1
NCGC00389063-01		2.3	744.4	34.5
NCGC00165840-02		>30	294.2	16.7
NCGC00420692-01		5	222.3	46.7
NCGC00478985-01		4.7	204.3	30.8
NCGC00478984-01		11.8	175.5	31.3
NCGC00475747-01		5.2	166.7	27.4
NCGC00475748-01		>30	138.8	>51
NCGC00167328-04	E64d	1.5	89	24
NCGC00484012-01	CA074	>30	<1	>77
NCGC00420691-01		6	75.9	50.1
NCGC00420693-01		4.1	67.3	55
NCGC00478986-01		6.2	62.3	29.8
NCGC00488908-01		>30	7.6	>60
NCGC00481260-01		54.5	11.8	>49
NCGC00408977-01		>30	8.4	>51
NCGC00384435-05		31.2	7.8	>49
NCGC00481259-01		>30	1.2	>49
NCGC00384435-04		31.6	9	>49
NCGC00384435-02		22.5	16.5	>49
NCGC00476231-01		>30	12.3	>47
NCGC00390171-01		>30	5.6	>45
NCGC00389061-01		2.5	28.2	>45
NCGC00476230-01		23.6	12.9	>44
NCGC00420690-01		6.3	31.7	40
NCGC00484094-01	CA074me	11.1	33	22
NCGC00023595-05	E64c	>30	<1	33
NCGC00488906-01		>30	ND	15.9
NCGC00487434-01		>30	ND	22
NCGC00389074-01		1.5	ND	20.7
NCGC00488907-01		6.4	<4.3	

## References

[1] Reichert E., Clase A., Bacetty A., Larsen J. (2009). Alphavirus antiviral drug development: Scientific gap analysis and prospective research areas. Biosecur. Bioterror..

[2] Sagripanti J.L., Rom A.M., Holland L.E. (2010). Persistence in darkness of virulent alphaviruses, Ebola virus, and Lassa virus deposited on solid surfaces. Arch. Virol..

[3] Hu X., Compton J.R., Leary D.H., Olson M.A., Lee M.S., Cheung J., Ye W., Ferrer M., Southall N., Jadhav A. (2016). Kinetic, Mutational, and Structural Studies of the Venezuelan Equine Encephalitis Virus Nonstructural Protein 2 Cysteine Protease. Biochemistry.

[4] Division of Vector-Borne Diseases (2021). National Center for Emerging and Zoonotic Infectious Diseases (NCEZID). https://www.cdc.gov/easternequineencephalitis/statistics-maps/historic-data.html.

[5] Gardner C.L., Burke C.W., Tesfay M.Z., Glass P.J., Klimstra W.B., Ryman K.D. (2008). Eastern and Venezuelan equine encephalitis viruses differ in their ability to infect dendritic cells and macrophages: Impact of altered cell tropism on pathogenesis. J. Virol..

[6] Maclachlan, James N., Dubovi E.J. (2017). Fenner’s Veterinary Virology.

[7] Pittman P.R., Makuch R.S., Mangiafico J.A., Cannon T.L., Gibbs P.H., Peters C.J. (1996). Long-term duration of detectable neutralizing antibodies after administration of live-attenuated VEE vaccine and following booster vaccination with inactivated VEE vaccine. Vaccine.

[8] Pratt W.D., Gibbs P., Pitt M.L., Schmaljohn A.L. (1998). Use of telemetry to assess vaccine-induced protection against parenteral and aerosol infections of Venezuelan equine encephalitis virus in non-human primates. Vaccine.

[9] Jahrling P.B., Stephenson E.H. (1984). Protective efficacies of live attenuated and formaldehyde-inactivated Venezuelan equine encephalitis virus vaccines against aerosol challenge in hamsters. J. Clin. Microbiol..

[10] Strauss E.G., Strauss J.H., Rawlings N.D., Salvesen G. (2013). Handbook of Proteolytic Enzymes.

[11] Morazzani E.M., Compton J.R., Leary D.H., Berry A.V., Hu X., Marugan J., Glass P.J., Legler P.M. (2019). Proteolytic cleavage of host proteins by the Group IV viral proteases of Venezuelan equine encephalitis virus and Zika virus. Antivir. Res..

[12] Zhou Z., Jia X., Xue Q., Dou Z., Ma Y., Zhao Z., Jiang Z., He B., Jin Q., Wang J. (2014). TRIM14 is a mitochondrial adaptor that facilitates retinoic acid-inducible gene-I-like receptor-mediated innate immune response. Proc. Natl. Acad. Sci. USA.

[13] Doctor K.Z., Gilmour E., Recarte M., Beatty T.R., Shifa I., Stangel M., Schwisow J., Leary D.H., Legler P.M. (2023). Automated SSHHPS Analysis Predicts a Potential Host Protein Target Common to Several Neuroinvasive (+)ssRNA Viruses. Viruses.

[14] Falk M.M., Grigera P.R., Bergmann I.E., Zibert A., Multhaup G., Beck E. (1990). Foot-and-mouth disease virus protease 3C induces specific proteolytic cleavage of host cell histone H3. J. Virol..

[15] Grigera P.R., Tisminetzky S.G. (1984). Histone H3 modification in BHK cells infected with foot-and-mouth disease virus. Virology.

[16] Reynolds N.D., Aceves N.M., Liu J.L., Compton J.R., Leary D.H., Freitas B.T., Pegan S.D., Doctor K.Z., Wu F.Y., Hu X. (2021). The SARS-CoV-2 SSHHPS Recognized by the Papain-like Protease. ACS Infect. Dis..

[17] Lim B.-K., Peter A.K., Xiong D., Narezkina A., Yung A., Dalton N.D., Hwang K.-K., Yajima T., Chen J., Knowlton K.U. (2013). Inhibition of Coxsackievirus-associated dystrophin cleavage prevents cardiomyopathy. J. Clin. Investig..

[18] Hu X., Compton J.R., Legler P.M. (2019). Analysis of Group IV Viral SSHHPS Using In Vitro and In Silico Methods. J. Vis. Exp..

[19] Smith E.C., Sexton N.R., Denison M.R. (2014). Thinking Outside the Triangle: Replication Fidelity of the Largest RNA Viruses. Annu. Rev. Virol..

[20] Dahlin J.L., Nissink J.W., Strasser J.M., Francis S., Higgins L., Zhou H., Zhang Z., Walters M.A. (2015). PAINS in the assay: Chemical mechanisms of assay interference and promiscuous enzymatic inhibition observed during a sulfhydryl-scavenging HTS. J. Med. Chem..

[21] Ruge D.R., Dunning F.M., Piazza T.M., Molles B.E., Adler M., Zeytin F.N., Tucker W.C. (2011). Detection of six serotypes of botulinum neurotoxin using fluorogenic reporters. Anal. Biochem..

[22] Kitz R., Wilson I.B. (1962). Esters of methanesulfonic acid as irreversible inhibitors of acetylcholinesterase. J. Biol. Chem..

[23] Murshudov G.N., Vagin A.A., Dodson E.J. (1997). Refinement of macromolecular structures by the maximum-likelihood method. Acta Crystallogr. Sect. D Biol. Crystallogr..

[24] Brunger A.T., Adams P.D., Clore G.M., DeLano W.L., Gros P., Grosse-Kunstleve R.W., Jiang J.S., Kuszewski J., Nilges M., Pannu N.S. (1998). Crystallography & NMR system: A new software suite for macromolecular structure determination. Acta Crystallogr. D Biol. Crystallogr..

[25] Emsley P., Lohkamp B., Scott W.G., Cowtan K. (2010). Features and development of Coot. Acta Crystallogr. D Biol. Crystallogr..

[26] Spurgers K.B., Hurt C.R., Cohen J.W., Eccelston L.T., Lind C.M., Lingappa V.R., Glass P.J. (2013). Validation of a cell-based ELISA as a screening tool identifying anti-alphavirus small-molecule inhibitors. J. Virol. Methods.

[27] Russo A.T., White M.A., Watowich S.J. (2006). The crystal structure of the Venezuelan equine encephalitis alphavirus nsP2 protease. Structure.

[28] Zhu T., Cao S., Su P.C., Patel R., Shah D., Chokshi H.B., Szukala R., Johnson M.E., Hevener K.E. (2013). Hit identification and optimization in virtual screening: Practical recommendations based on a critical literature analysis. J. Med. Chem..

[29] Montaser M., Lalmanach G., Mach L. (2002). CA-074, but not its methyl ester CA-074Me, is a selective inhibitor of cathepsin B within living cells. Biol. Chem..

[30] Chevriaux A., Pilot T., Derangere V., Simonin H., Martine P., Chalmin F., Ghiringhelli F., Rebe C. (2020). Cathepsin B Is Required for NLRP3 Inflammasome Activation in Macrophages, Through NLRP3 Interaction. Front. Cell Dev. Biol..

[31] Deregnaucourt J., Andre E., Tisne-Versailles J. (2012). Use of Antihistamine Agents for the Preventive or Early Treatment of Inflammatory Syndromes, in Particular Those Triggered by Togaviruses. U.S. Patent.

[32] Schroeder C.E., Yao T., Sotsky J., Smith R.A., Roy S., Chu Y.K., Guo H., Tower N.A., Noah J.W., McKellip S. (2014). Development of (E)-2-((1,4-dimethylpiperazin-2-ylidene)amino)-5-nitro-N-phenylbenzamide, ML336: Novel 2-amidinophenylbenzamides as potent inhibitors of venezuelan equine encephalitis virus. J. Med. Chem..

[33] Lee J., Parvathareddy J., Yang D., Bansal S., O’Connell K., Golden J.E., Jonsson C.B. (2020). Emergence and Magnitude of ML336 Resistance in Venezuelan Equine Encephalitis Virus Depend on the Microenvironment. J. Virol..

[34] Chung D., Schroeder C.E., Sotsky J., Yao T., Roy S., Smith R.A., Tower N.A., Noah J.W., McKellip S., Sosa M. (2010). ML336: Development of Quinazolinone-Based Inhibitors against Venezuelan Equine Encephalitis Virus (VEEV). Probe Reports from the NIH Molecular Libraries Program.

[35] Pluimer B.R., Colt M., Zhao Z. (2020). G Protein-Coupled Receptors in the Mammalian Blood-Brain Barrier. Front. Cell. Neurosci..

[36] Chang J., Mancuso M.R., Maier C., Liang X., Yuki K., Yang L., Kwong J.W., Wang J., Rao V., Vallon M. (2017). Gpr124 is essential for blood-brain barrier integrity in central nervous system disease. Nat. Med..

[37] Paessler S., Aguilar P., Anishchenko M., Wang H.-Q., Aronson J., Campbell G., Cararra A.-S., Weaver S.C. (2004). The Hamster as an Animal Model for Eastern Equine Encephalitisb. J. Infect. Dis..

